# Delta-like 3 is silenced by HBx via histone acetylation in HBV-associated HCCs

**DOI:** 10.1038/s41598-018-23318-1

**Published:** 2018-03-19

**Authors:** Hiroki Hamamoto, Kentaro Maemura, Kentaro Matsuo, Kohei Taniguchi, Yoshihisa Tanaka, Sugiko Futaki, Atsushi Takeshita, Akira Asai, Michihiro Hayashi, Yoshinobu Hirose, Yoichi Kondo, Kazuhisa Uchiyama

**Affiliations:** 1 Departments of General and Gastroenterological Surgery, Osaka Medical College, Takatsuki, 569-8686 Japan; 2 Departments of Anatomy and Cell Biology, Osaka Medical College, Takatsuki, 569–8686 Japan; 3 Departments of Pathology, Osaka Medical College, Takatsuki, 569–8686 Japan; 4 Second Department of Internal Medicine, Osaka Medical College, Takatsuki, 569–8686 Japan

## Abstract

Hepatocellular carcinoma (HCC) is a common malignant tumor with poor prognosis. We previously showed that expression of Delta-like 3 (DLL3), a member of the family of Delta/Serrate/Lag2 ligands for the Notch receptor, is silenced by aberrant DNA methylation and that overexpression of DLL3 in an HCC cell line induces cellular apoptosis. However, how DLL3 expression is regulated during hepatocarcinogenesis is still unclear. Here, we show that silencing of DLL3 during hepatocarcinogenesis is closely related to viral infection, especially hepatitis B virus (HBV) infection (p = 0.005). HepG2.2.15 cells, which are stably transformed with the HBV genome, showed lower DLL3 expression than the parent cell line, HepG2 cells. Treatment with Hepatitis B virus X protein (HBx) small interfering RNA upregulated DLL3 expression in HepG2.2.15 cells, and overexpression of HBx in HepG2 cells downregulated DLL3 expression. Treatment of cells with a histone deacetylase inhibitor induced DLL3 expression in HepG2.2.15 cells. These data suggest that DLL3 expression is silenced during hepatocarcinogenesis in association with HBV infection via an epigenetic mechanism.

## Introduction

Hepatocellular carcinoma (HCC) is a common malignant tumor with poor prognosis. HCC is frequently caused by chronic hepatitis B virus (HBV) or hepatitis C virus (HCV) infection, alcohol abuse, non-alcoholic steatohepatitis, exposure to aflatoxin B1, and hemochromatosis^1^. The precise molecular mechanisms that mediate HCC development are still unclear, but many studies have revealed that hepatocarcinogenesis is a multistep process that includes activation of oncogenes and inactivation of tumor suppressor genes due to aberrant genetic and epigenetic events^2–4^. Regarding genetic aberrations, Fujimoto *et al*.^5^ reported that chromatin regulation genes such as AT-Rich Interaction Domain 1A (*ARID*), *ARID1B*, *ARID2*, myeloid/lymphoid or mixed-lineage leukemia (*MLL*), and *MLL3* contain many mutations. Mutations in tumor protein p53 (*TP53*) activate the WNT/beta-catenin pathway and inactivate retinoblastoma and the insulin-like growth factor 2 pathway in HCC^6^.

Epigenetic mechanisms such as DNA methylation, histone modifications, chromatin remodeling, and non-coding RNAs regulate gene expression via multiple types of cross-talk^7,8^. DNA methylation is one of the most widely studied epigenetic mechanisms. In mammals, DNA methylation is the covalent addition of a methyl group to the C-5 position of cytosine by DNA methyltransferases to form 5-methylcytosine. This reaction occurs almost exclusively on cytosine residues of CpG dinucleotides.

However, CpG islands, which are regions with a relatively high content of CpG dinucleotides, are generally associated with promoters of genes that are expressed in most tissues^9^. Aberrant DNA methylation of promoter CpG islands is associated with silencing of tumor suppressor genes in a variety of human cancers^10–12^. Sun *et al*.^11^ reported that cyclooxygenase2 (*COX2*), p16, Ras association domain family 1 isoform A (*RASSF1A*), and tissue inhibitor of metalloproteinases3 (*TIMP-3*) are frequently methylated in HCC, but not in non-cancerous liver tissues. Yoshikawa *et al*.^13^ conducted restriction landmark genomic scanning analysis and isolated several aberrantly methylated genes such as suppressor of cytokine signaling-1 (*SOCS-1*), *SOCS-3*, and apoptotic speck protein-like (*ASCL*) in HCC.

Histone acetylation also plays a key role in epigenetic regulation by changing the structure of chromatin, thus modulating the accessibility of transcription factors to their target DNA^14^. Histone acetylation is higher in transcriptionally active regions of several cancer-related genes such as *TP53*, core binding factor α3 (*RUNX3*), β-catenin, Avian myelocytomatosis viral oncogene homolog (*Myc*), and others^15^.

Notch signaling is triggered by binding of a transmembrane ligand to the Notch transmembrane receptor on a neighboring cell. This results in proteolytic cleavage of the Notch receptor, releasing the constitutively active Notch intracellular domain (NICD). NICD translocates to the nucleus and regulates downstream transcriptional gene expression^16^. Notch signaling is essential for various biological processes such as differentiation, proliferation, and apoptosis^16,17^.

Delta-like 3 (DLL3) is a member of the Delta/Serrate/Lag2 family of ligands for Notch receptors. DLL3 is the most structurally different of these ligands and is not located on the cell membrane, but in the Golgi apparatus^18^. Ladi *et al*.^19^ reported that DLL3 does not activate Notch signaling and does not bind to cells that express Notch receptors. Therefore, DLL3 is considered an inhibitor of Notch signaling.

Loomes *et al*.^20^ reported that DLL3 is highly expressed in fetal brain, and the mRNA is expressed at lower levels in human adult liver^21^. We previously showed that DLL3 is frequently methylated in HCC cell lines, and overexpression of DLL3 in HCC cell lines induces apoptosis^22^. However, the role of DLL3 in hepatocarcinogenesis is not clear. Thus, the aim of this study is to clarify the regulation of DLL3 expression during hepatocarcinogenesis. To achieve this, we first investigated DLL3 expression in surgically resected HCC specimens and adjacent liver and analyzed the clinicopathological factors that affect DLL3 expression. In addition, we investigated the regulation of DLL3 expression by HBV using HCC cell lines.

## Results

### DLL3 expression in normal liver

**Figure 1:**
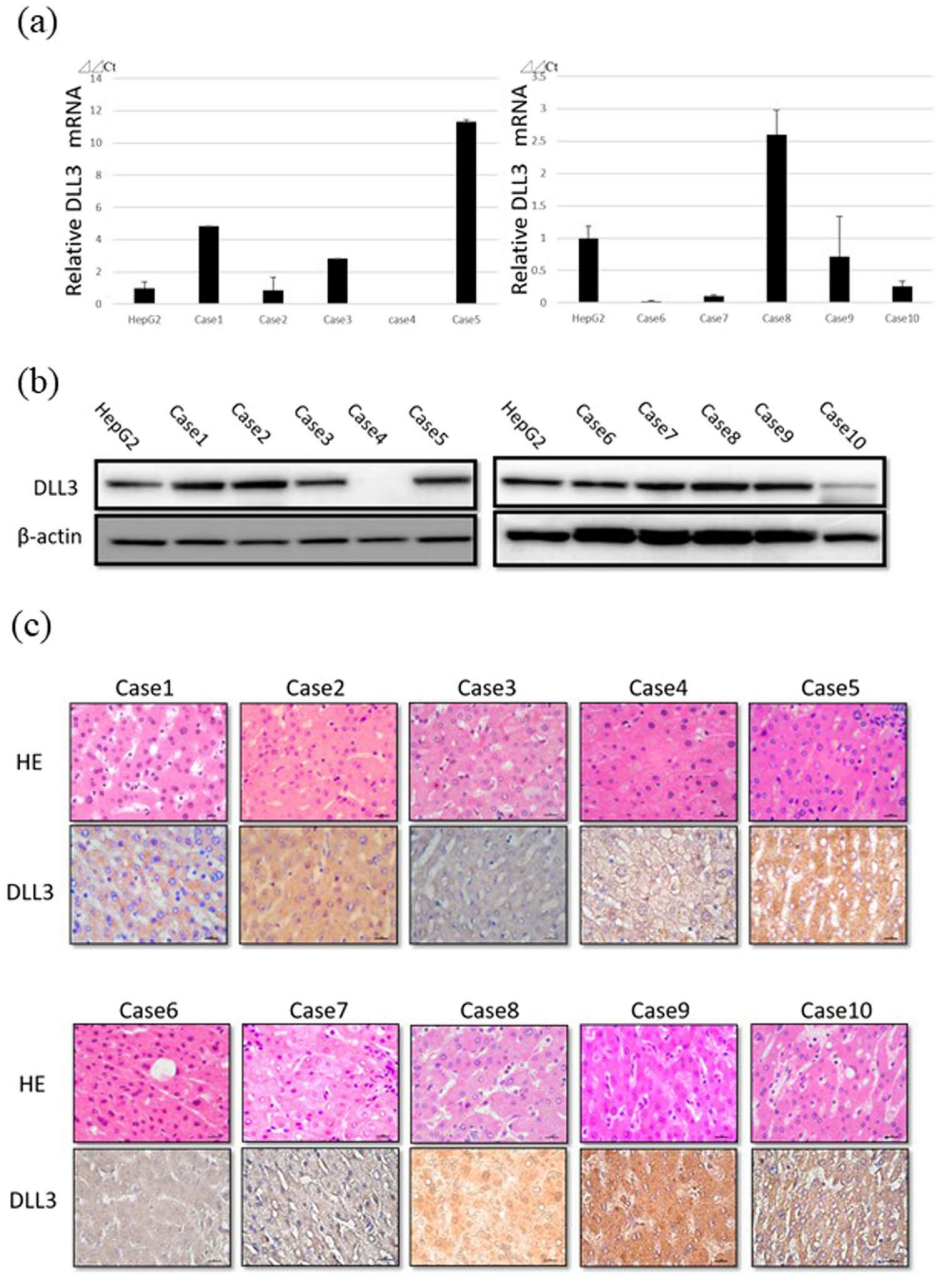
DLL3 expression in normally functioning livers. (**a**) The relative quantity of *DLL3* mRNA in normally functioning livers was evaluated with qRT-PCR. The HepG2 cell line was used as a positive control. (**b**) DLL3 was detected with western blot analysis under the same experimental conditions at the same time. β-actin was used as a loading control. (**c**) Immunohistochemical staining of DLL3 protein. Positive signals were detected in the cytoplasm of hepatocytes. Scale bar, 10 μm.

We examined 10 liver specimens from patients with colorectal cancer or rectal carcinoid metastasis to the liver. DLL3 expression in normal liver was evaluated with quantitative real-time polymerase chain reaction (qRT-PCR), western blot analysis, and immunohistochemistry. As shown in Supplementary Table S1, preoperative laboratory data for 10 patients were almost normal. DLL3 expression was observed in nine of 10 samples of normally functioning livers (90%) as seen with qRT-PCR, western blot analysis (Fig. 1a,b, Supplementary Figure S2), and immunohistochemistry (Fig. 1c). Neither fibrosis nor steatosis were observed following hematoxylin and eosin staining (Fig. 1c). In this analysis of normal liver, we used HepG2 cells as a positive control and confirmed DLL3 expression in preliminary experiments (data not shown).

### DLL3 expression in HCCs

We next examined liver specimens from 46 additional patients with HCC. The clinicopathological features of these 46 HCC patients are summarized in Supplementary Table S3. The specimens prepared from nine of these HCC patients included severe tumor necrosis, and thus, tissues from only 37 HCC patients were subjected to immunohistochemistry. As shown in Table 1, in cases in which the tumor diameter was less than 5 cm, DLL3 expression was significantly lower (p = 0.0375) than in larger tumors. Low DLL3 expression was confirmed in 22 of 23 (95.6%) HCCs in which the size was less than 5 cm, and in 10 of 14 (71.3%) HCCs in which the size was greater than 5 cm.

**Table 1:** DLL3 expression in HCCs.

	DLL3 expression		p
	High (>50%, IHC)	Low (≦50%, IHC)	
Gender			
Male	4	24	0.8050
Female	1	8	
Age			
<65	1	17	0.1547
≥65	4	15	
Differentiation			
Well-Mod	5	30	0.8227
Poor	0	2	
Tumor size			
<5 cm	1	22	0.0375^*^
≥5 cm	4	10	
T classification (UICC)			
T1-2	2	13	0.9789
T3-4	3	19	
Stage (UICC)			
I–II	2	13	0.9789
III–IV	3	19	

UICC = The Union of International Cancer Control.

^*^P < 0.05.

### DLL3 expression in adjacent non-cancerous liver specimens

**Figure 2:**
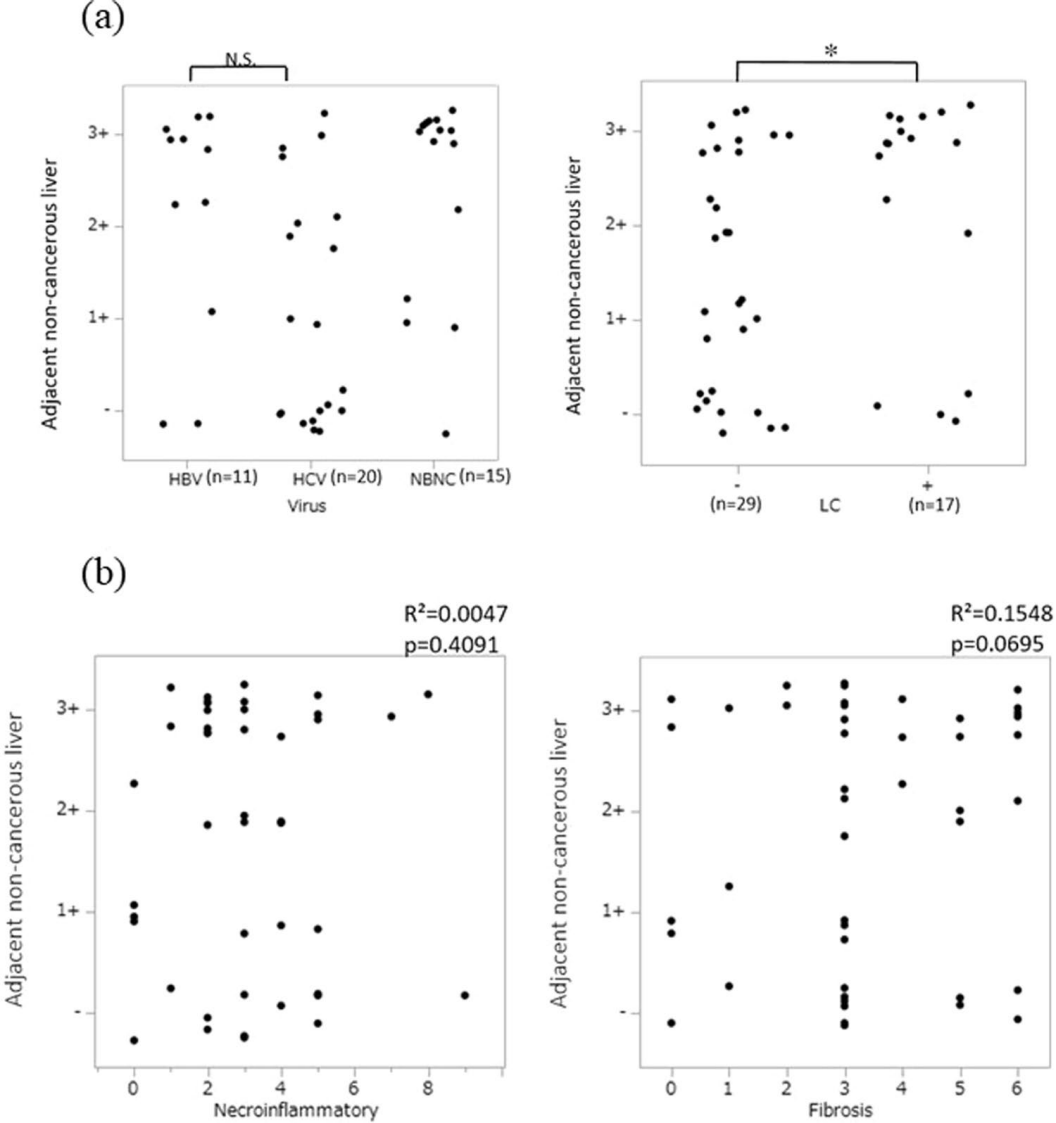
The relationship between DLL3 expression in non-cancerous liver and clinicopathological features. (**a**) The relationship between hepatitis virus infection and DLL3 expression scores from immunohistochemical staining. LC = Liver cirrhosis. (**b**) The relationship between necroinflammation scores and DLL3 expression, and between fibrosis scores and DLL3 expression. (*p < 0.05; N.S. = not statistically significant).

Expression of DLL3 in 46 non-cancerous liver tissues adjacent to HCC from the above 46 patients was analyzed with immunohistochemistry. In non-cancerous liver specimens, no difference was found in the expression of DLL3 in patients with hepatitis B and hepatitis C (Fig. 2a (left)). As shown in Fig. 2a (right), strong DLL3 expression was observed in non-cancerous cirrhotic liver compared to non-cirrhotic liver (p = 0.0255). Strong DLL3 expression (3+) was observed in 11 of the 17 cirrhotic liver cases (64.7%) and in nine of the 29 non-cirrhotic liver cases (31.0%). Next, we investigated the relationship between necroinflammation or fibrosis scoring using the Ishak scoring system and DLL3 expression in non-cancerous liver tissues adjacent to HCC. As shown in Fig. 2b (right), tissues with severe fibrosis tended to have stronger DLL3 expression, although no significant correlation was observed (Fig. 2b (right), R^2^ = 0.1548, p = 0.0695). We found no significant correlation between the necroinflammatory score and DLL3 expression (Fig. 2b (left), R^2^ = 0.0047, p = 0.4091). The results of the Ishak scores are summarized in Supplementary Table S4.

### Silencing of DLL3

**Figure 3:**
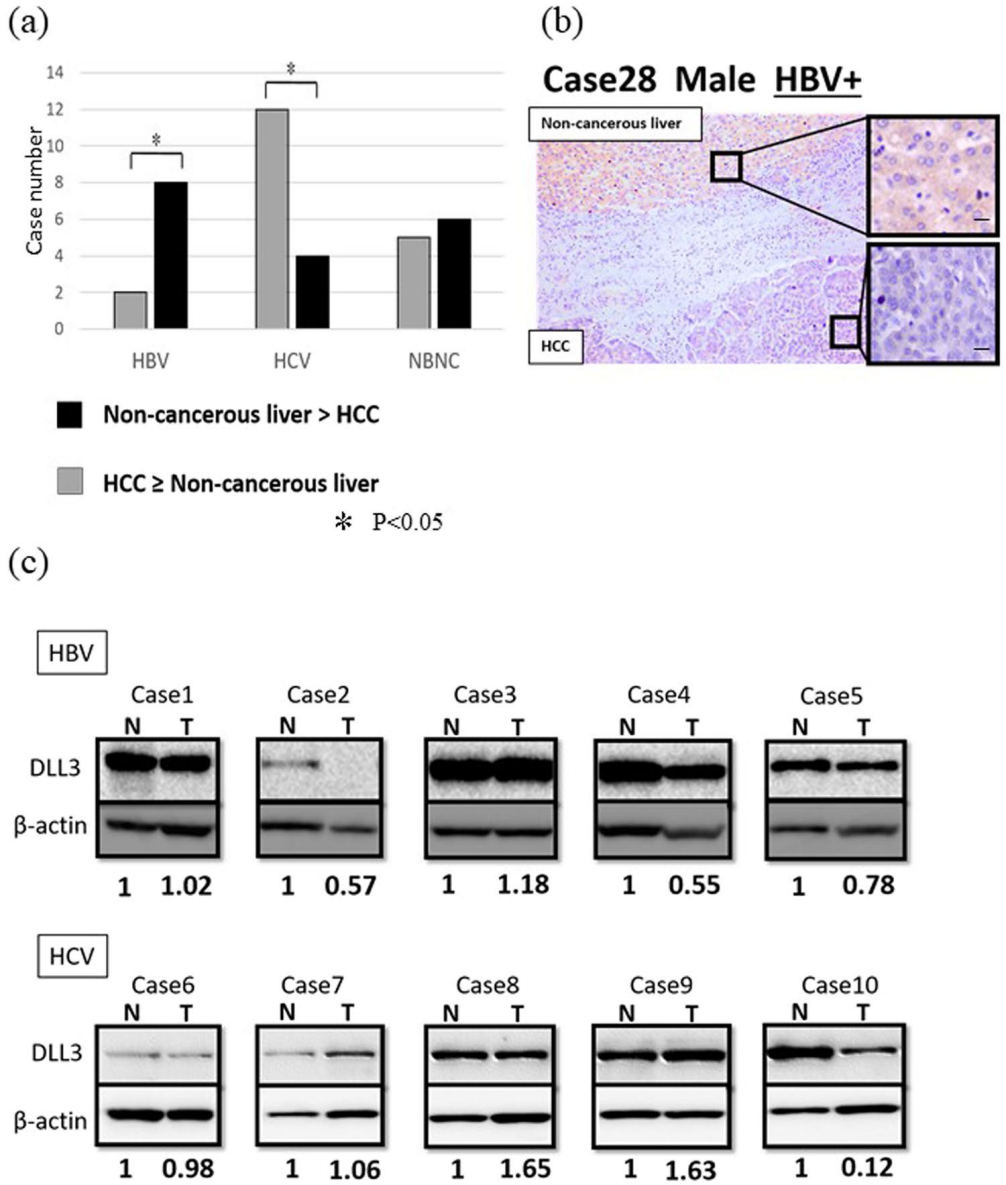
DLL3 silencing in HBV-associated HCCs. (**a**) Analysis of the relative expression of DLL3 in 37 paired HCC and non-cancerous liver specimens. (*p < 0.05) (**b**) Immunohistochemical staining of DLL3 protein in a representative HCC and non-cancerous liver specimen. DLL3 was expressed at lower levels in HCC compared to non-cancerous liver. Scale bar, 10 μm. (**c**) The protein expression of DLL3 in clinical specimens of HCC (T) and non-cancerous liver (N). Cases 1–5 were HBV-associated HCC and paired non-cancerous liver, and Cases 6–10 were HCV-associated HCC and paired non-cancerous liver. β-actin was used as a loading control. The numbers under bands showed the relative level of signal when the signals of non-cancerous liver (N) were used as a control. Densitometric values of DLL3 were calculated with Fusion FX.

We used immunohistochemistry to investigate whether HBV or HCV infection affects the silencing of DLL3 expression in HCC. As shown in Fig. 3a, silencing of DLL3 was frequently observed in HBV-associated HCCs (Fig. 3a, p = 0.005). Silencing of DLL3 was confirmed in eight of the 10 cases (80%) of HBV-associated HCCs and in four of the 16 cases (25%) of HCV-associated HCCs. Immunohistochemical staining of DLL3 protein in a representative HCC and non-cancerous liver specimen is shown in Fig. 3b. DLL3 was expressed at lower levels in HCC compared to non-cancerous liver. Next, we performed immunocytochemistry to evaluate the expression of Hepatitis B virus X protein (HBx), a functional protein produced by HBV, in 10 patients with HBV-associated HCCs and adjacent non-cancerous livers. Very strong signals were detected in all HCCs and non-cancerous livers. We found no difference in HBx expression between HCCs and non-cancerous livers (Supplementary Figure S5). Western blot analysis showed that silencing of DLL3 was present in three of five cases (60%: cases 2, 4, and 5) of HBV-associated HCCs and in one of five cases (20%: case 10) of HCV-associated HCCs (Fig. 3c, Supplementary Figure S6). In this western blot analysis, β-actin signals varied among cases, and thus, we calculated densitometric values of DLL3 with respect to β-actin for each case. These results suggest that silencing of DLL3 expression is associated with HBV infection.

### DLL3 expression in HepG2 and HepG2.2.15 cells

**Figure 4:**
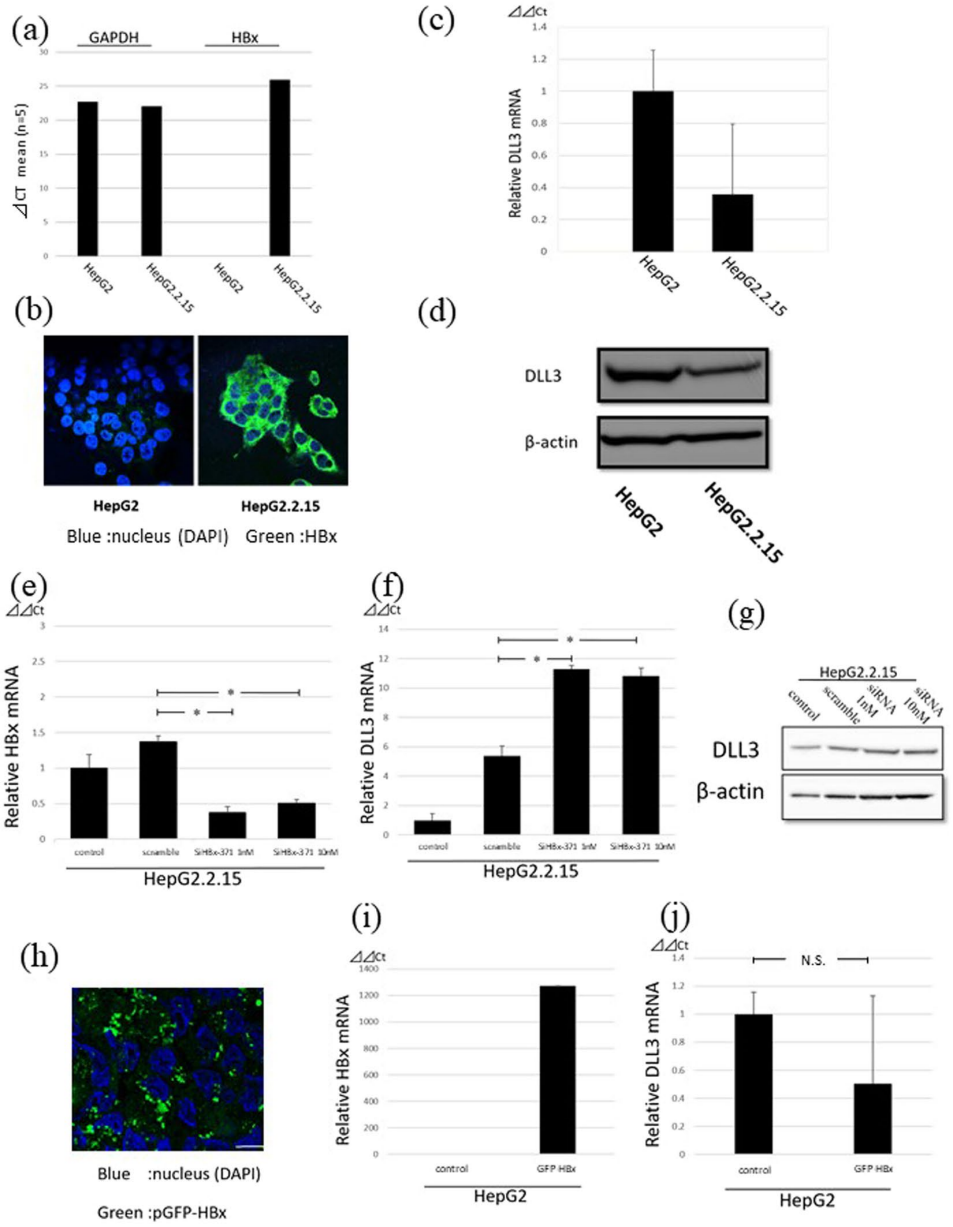
HBx suppressed DLL3 expression. (**a**) Relative quantity of *HBx* mRNA in HepG2 and HepG2.2.15 cells was evaluated with qRT-PCR. *HBx* amplification in HepG2 cells was not observed. (**b**) HBx expression in HepG2 and HepG2.2.15 cells was evaluated with immunocytochemistry. Scale bar, 10 μm. (**c**,**d**) Relative quantity of *DLL3* mRNA and protein in HepG2 and HepG2.2.15 cells was evaluated with qRT-PCR (**c**) and western blot analysis (**d**), respectively. (**e**) Relative quantity of *HBx* mRNA in HepG2.2.15 cells treated with siRNA was evaluated with qRT-PCR. (**f**,**g**) *HBx* expression in HepG2.2.15 cells treated with siRNA was evaluated with qRT-PCR (**f**) and western blot analysis (**g**,**h**) Successful transfection of pGFP-HBx was confirmed with immunocytochemistry. Scale bar, 10 μm. (**i**,**j**) Relative quantity of *HBx* (**i**) and *DLL3* (**j**) mRNA in HepG2.2.15 cells transfected with pGFP-HBx was evaluated with qRT-PCR. (N.S. = not statistically significant).

To confirm whether HBV infection plays a role in the silencing of DLL3 expression, HepG2 and HepG2.2.15 cell lines were used in further experiments. HepG2.2.15 cells are stably transformed with the HBV genome^23^. The expression of HBx was confirmed with qRT-PCR and immunocytochemistry in HepG2.2.15 cells, whereas no HBx expression was observed in the parent HepG2 cells (Fig. 4a,b). Real-time PCR and western blot analysis demonstrated that DLL3 was expressed in HepG2 cells and that the level of expression in HepG2.2.15 cells was lower than in HepG2 cells (Fig. 4c,d, Supplementary Figure S7a).

### Knockdown of HBx

Gene silencing was performed to investigate the effects of HBx on DLL3 expression.

Two types of HBx small interfering RNA (siRNA) (siHBx-260 and siHBx-371) were prepared. siHBx-371 was used in further experiments because it suppressed HBx expression in HepG2.2.15 cells more strongly (Supplementary Figure S8). Successful knockdown of HBx was confirmed (Fig. 4e). We evaluated the siRNA transfection efficiency using fluorescent microscopy with fluorescein-tagged siHBx-371 (data not shown). siHBx-371 (1 nM or 10 nM) increased both DLL3 mRNA and DLL3 protein expression in HepG2.2.15 cells (Fig. 4f,g, Supplementary Figure S7b).

### Overexpression of HBx

Further, we evaluated the role of HBx in DLL3 expression by transfecting HepG2 cells with an HBx expression vector. First, we determined the transfection conditions by observing transfected cells under a fluorescent microscope. Around 80% of the cells expressed HBx, and *HBx* mRNA expression was induced by transfecting cells with the plasmid (Fig. 4h,i). As shown in Fig. 4j, expression of *DLL3* mRNA was downregulated following transfection of the expression vector, although the difference was not significant compared to the control. These data using cell lines suggest that DLL3 expression is downregulated in HBV-associated HCC via HBx.

### Treatment with 5-azadeoxycitidine (5-Aza-dC) and trichostatin A (TSA)

**Figure 5:**
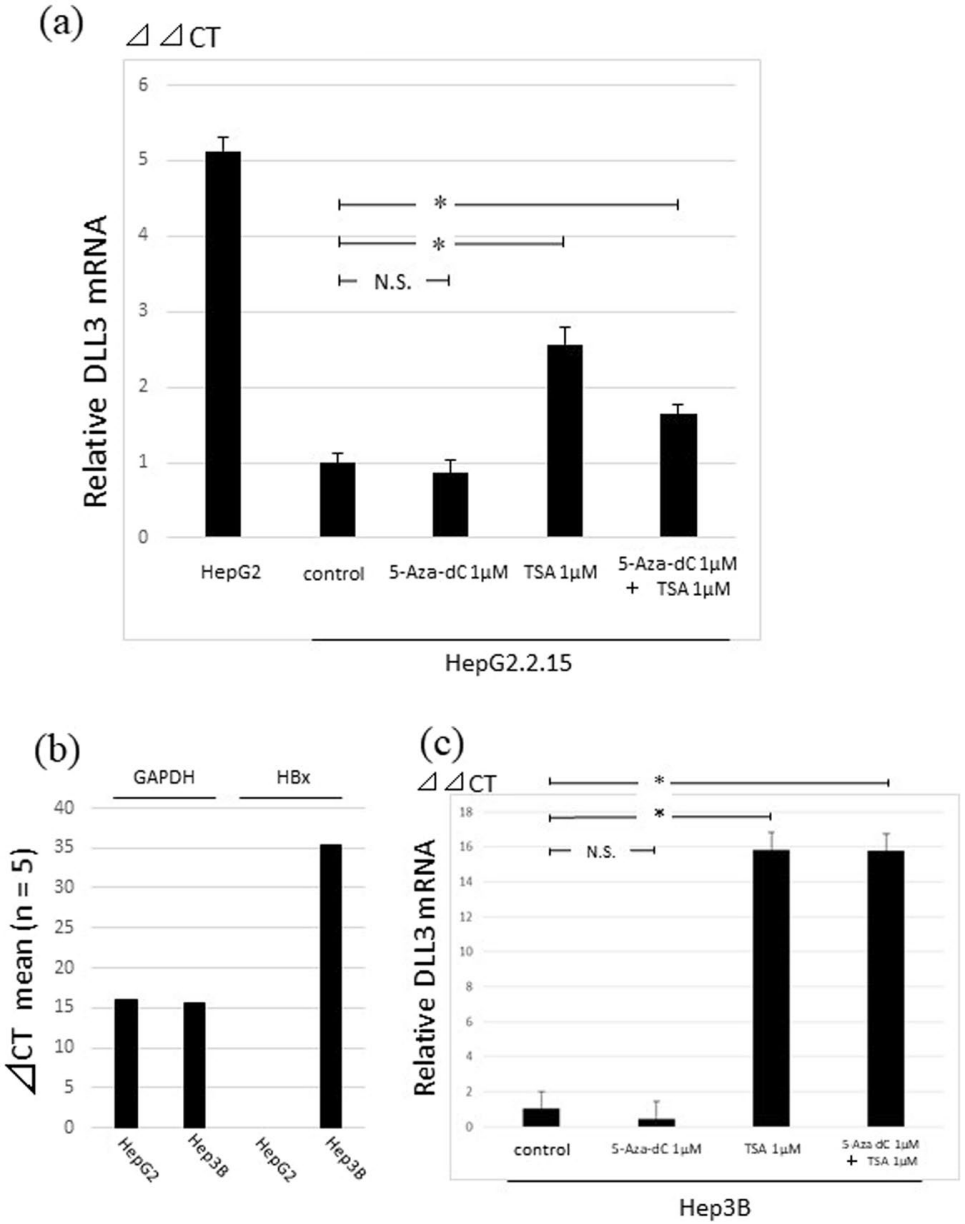
DLL3 silencing via histone acetylation in hepatocellular cell lines. (**a**) Relative quantity of *DLL3* mRNA in HepG2.2.15 cells treated with 1 µM 5-Aza-dC, 1 µM TSA, or 1 µM 5-Aza-dC +1 µM TSA. (*p < 0.05; N.S. = not statistically significant). (**b**) Relative quantity of *HBx* mRNA in Hep3B cells was detected with qRT-PCR. (**c**) Relative quantity of *DLL3* mRNA in Hep3B cells treated with 1 µM 5-Aza-dC, 1 µM TSA, or 1 µM 5-Aza-dC +1 µM TSA. (*p < 0.05; N.S. = not statistically significant).

HBx is a transactivator of multiple cellular promoters, which interact with DNA methyltransferase 3 A or recruit histone deacetylase (HDAC). Thus, we investigated the effect of a DNA methylation inhibitor or HDAC inhibitor on DLL3 expression in HepG2.2.15 and Hep3B cells. As shown in Fig. 5a, TSA, which is an HDAC inhibitor, upregulated *DLL3* mRNA expression by 3-fold, but no effect was observed following 5-Aza-dC treatment. The expression of HBx was confirmed with qRT-PCR in Hep3B cells (Fig. 5b). TSA also upregulated *DLL3* mRNA expression in Hep3B cells, but 5-Aza-dC did not (Fig. 5c).

## Discussion

Previous studies have shown that DLL3 plays important roles in embryonic development and that the function of DLL3 in Notch signaling is different from other Notch ligands^18^. Because DLL3 is rarely expressed on the cell surface, it cannot *trans*-activate Notch receptors on neighboring cells. However, DLL3 can *cis*-inhibit Notch signaling activated by DLL1^19^.

DLL3 is highly expressed in human renal cancer and high-grade pulmonary neuroendocrine tumors^21,24^. However, the roles of DLL3 in carcinogenesis have remained unclear. Saunders *et al*.^21^ reported that a DLL3-targeted antibody-drug conjugate (Rovalpituzumab tesirine, Rova-T) induces tumor regression in high-grade pulmonary neuroendocrine tumors such as small cell lung cancer (SCLC) and large cell neuroendocrine carcinoma. According to their microarray data, the *DLL3* mRNA level in normal liver is low. However, our previous and present study showed that DLL3 is expressed in normal, steatotic, and fibrotic liver tissues^25^. The background of their microarray tissue is unclear, and thus, we believe that our data are more reliable. Additionally, as shown in Fig. 1a,b, the mRNA levels and protein levels of DLL3 were not always consistent. The discrepancy between the mRNA levels and protein levels of DLL3 suggested the possibility that DLL3 expression is regulated by unknown factors at the translation stage. DLL3 is expressed in more than 80% of patients with SCLC^26^. A phase 1 study of Rova-T in recurrent SCLC showed that the drug is safe and effective^26^. In their study, liver dysfunction due to side effects occurred in only one of 74 patients (1%). Our immunohistochemical study showed that DLL3 is expressed in the cytoplasm of hepatocytes, whereas DLL3 is expressed on the cell surface in SCLC. Thus, we assume that no serious liver dysfunction will occur after Rova-T treatment.

We previously reported that DLL3 expression is silenced by DNA methylation in several HCC cell lines^22^. So far, no studies have reported DLL3 expression in clinical HCC tissues. In the present study, we investigated the relationship between DLL3 expression and clinicopathological factors using surgically resected HCC tissues. Our immunohistochemical study showed that DLL3 staining was significantly elevated in non-cancerous cirrhotic livers. In addition, the degree of fibrosis as determined by the Ishak score was positively correlated with DLL3 expression. Patients whose Ishak score is too high may not be surgical candidates, and thus, patients with serious liver dysfunction were excluded from this study. Further studies are necessary to confirm the correlation between DLL3 expression and the pathological degree of chronic hepatitis.

Our immunohistochemical study showed that DLL3 expression was silenced in HBV-associated HCCs, and western blot analysis supported the results. HBV infection is the major etiological factor for HCC, but the molecular mechanisms underlying HBV-associated HCCs remain to be fully clarified. We evaluated HBx expression in HBV-associated HCCs and non-cancerous livers, and observed no difference between them. Many studies have revealed that HBx protein, which is essential for virus replication *in vivo*, plays a critical role in hepatocarcinogenesis^27^. HBx interrupts liver cell apoptosis and DNA repair mechanisms by modulating the transcriptional activation of p53^28^. HBx also causes mitochondrial injury by downregulating mitochondrial enzymes involved in electron transport for oxidative phosphorylation^29^.

As shown in Fig. 4c,d, DLL3 expression was significantly lower in HepG2.2.15 cells, which are transformed with the HBV genome, compared to parent HepG2 cells.

HBx knockdown induced upregulation of DLL3 expression, and overexpression of HBx in HepG2 cells tended to suppress DLL3 expression. HBx suppresses the expression of tumor-suppressor genes such as *RASSF1A* and *SOCS-1*^30,31^. We previously reported that DLL3 induces apoptosis in HCCs^22^, and this effect may be regulated by HBx.

HBx induces not only genetic modifications but also epigenetic modifications in HCCs^27^. Regarding epigenetic modifications, HBx induces DNA hypermethylation of host genes related to tumor suppression, DNA hypomethylation of tumor promotion-related genes, and histone acetylation/deacetylation of tumor-associated genes. Among these, the acetylation status of histones is regulated by histone acetyltransferases and HDACs^32^. Herein, we showed that DLL3 expression was upregulated by an HDAC inhibitor in HepG2.2.15 cells.

Several HDAC inhibitors are currently in clinical trials both for solid and hematological malignancies^33^. The anticancer effect of HDAC inhibitors is due to transcriptional reactivation of host tumor suppressor genes. HDAC inhibitors also modulate expression of several other genes related to the cell cycle, apoptosis, and angiogenesis^15^. Many inhibitors are known, but the most potent discovered so far is TSA, which is a fermentation product of *Streptomyces*. Thus, TSA was first used as an anti-fungal agent, but was later discovered to inhibit both G1 and G2 phases of the mammalian cell cycle and to have potent inhibitory effects on proliferation of cancer cells^34^. TSA selectively inhibits enzymes that belong to the class I, II, and IV, but not class III, mammalian HDAC families. In the present study, we showed that DLL3 expression was upregulated following treatment with an HDAC inhibitor in two HBV-associated HCC cell lines. DLL3 expression was upregulated 3-fold in HepG2.2.15 cells following treatment with an HDAC inhibitor, but DLL3 expression was lower than in parent HepG2 cells. In our previous study^22^, we demonstrated DLL3 silencing by methylation in other HCC cell lines; however, DLL3 expression was not changed in HepG2.2.15 cells following treatment with 5-Aza-dC. Epigenetic regulation of DLL3 expression may vary depending on the HCC cell line. These results suggest that epigenetic mechanisms other than DNA methylation may be involved in DLL3 silencing.

Zekri *et al*.^35^ reported that DLL3 is upregulated in circulating CD133+ cells from HCC patients associated with HCV compared to cells from healthy control patients. CD133 is a surface marker of cancer stem cells, and thus, DLL3 upregulation in CD133+ peripheral cells may affect hepatocarcinogenesis in HCV-associated HCCs. Our results showed that DLL3 was silenced in HBV-associated HCCs and suppressed by HBx. Further studies will be needed to elucidate the influence of DLL3 on hepatocarcinogenesis in HBV-associated HCC compared to HCV-associated HCC.

In conclusion, we demonstrated that DLL3 was expressed in normal livers and non-cancerous cirrhotic livers. DLL3 silencing was frequently observed in HBV-associated HCC. Further, we provide evidence showing that DLL3 was silenced by HBx via histone acetylation in HBV-associated HCCs. DLL3 potentially induces apoptosis, and thus, DLL3 may be a molecular target of HDAC inhibitors for HBV-associated HCCs.

## Methods

### Clinical specimens

Normal liver tissues were obtained from surgically resected tissues diagnosed as liver metastasis from colorectal cancer or rectal carcinoid. Ten patients with colorectal cancer or rectal carcinoid underwent hepatectomy in the Department of General and Gastroenterological Surgery, Osaka Medical College (Osaka, Japan). No patients had liver dysfunction, were infected with HBV or HCV, or were receiving oxaliplatin or irinotecan chemotherapy. Historical examination showed neither fibrosis nor steatosis in resected non-cancerous areas of the liver.

In a separate group of patients with primary HCC, paired HCC and adjacent non-cancerous liver tissues were obtained from residual liver tissues following surgery at the Department of General and Gastroenterological Surgery, Osaka Medical College. Forty-six samples were subjected to immunohistochemical analysis, and 10 of these samples were used for western bolt analysis. All samples were obtained after receiving written informed consent from the patients. The study was reviewed and approved by the institutional review board (IRB) of Osaka Medical College (IRB acceptance number: 1788) and was performed in accordance with the Declaration of Helsinki.

### Cell lines

The human HCC cell line HepG2 was purchased from the Japanese Collection of Research Bioresources Cell Bank. HepG2.2.15 was a gift from Dr. Tatsuo Kanda, Department of Gastroenterology and Nephrology, Chiba University. Hep3B was a gift from Nobuhiko Tanigawa (an emeritus professor of our department). Cell lines were maintained in RPMI-1640 medium (Sigma-Aldrich, St. Louis, Missouri, USA) containing 10% fetal bovine serum (Invitrogen Life Technologies, Waltham, Massachusetts, USA) at 37 °C in a 5% CO_2_ atmosphere.

### Immunohistochemical analysis

Immunohistochemistry was performed on 5-μm-thick formalin-fixed, paraffin-embedded tissue sections mounted on adhesive glass slides. The sections were deparaffinized with xylene and hydrated with a series of ethanol. Then, the specimens were pretreated with 10% bovine serum albumin in phosphate-buffered saline (PBS), and endogenous peroxidases were blocked by incubating the sections with 5 mM periodic acid (P-7875, Sigma-Aldrich) in distilled water. The tissue specimens were incubated with the anti-DLL3 polyclonal antibody (Funakoshi Co. Ltd., Japan, 1:1000) and anti-HBx monoclonal antibody (Abcam, Cambridge, UK, 1:1000) overnight at 4 °C. Signals were detected using Dako EnVision TM Dual Link System-HRP kit (1:1000) and 3,3′-diaminobenzidine staining, and sections were counterstained with hematoxylin at room temperature. The specimens were inspected and photographed with a microscope (Eclipse E600, Nikon, Japan).

Qualitative assessment of immunohistochemistry was done by estimation of the number of positively stained cells by two researchers (H.H. and K.M.) who were blinded to the background of patients. The HCC and adjacent non-cancerous liver sections were graded as follows: − (negative, no positively stained cells), + (positive, <20% positive cells), 2+ (strongly positive, 20–50% positive cells), 3+ (very strongly positive, ≥50% positive cells). Images were captured with the Nikon Eclipse E600 microscope system.

### Ishak score

The degree of inflammation and fibrosis in adjacent non-cancerous liver specimens was evaluated with the Ishak staging system, a modification of the Knodell system, which is the most widely accepted scoring system for the assessment of liver fibrosis and necroinflammation in chronic hepatitis^36^. Histopathological evaluation was carried out by a pathologist who specializes in the liver (T.A.).

### Western blot analysis

Tissue samples and cultured cells were homogenized in lysis buffer [10 mM Tris-HCl pH 7.4, 1% NP-40, 0.1% deoxycholic acid, 0.1% sodium dodecyl sulfate (SDS), 150 mM NaCl, 1 mM EDTA, 1% Protease Inhibitor Cocktail (Sigma-Aldrich)] and incubated on ice for 20 min. After centrifugation at 12,000 rpm for 20 min at 4 °C, the supernatants were collected as the total protein. Lysate proteins (10 µg) were separated by SDS-polyacrylamide gel (10.0%) electrophoresis and transferred onto PVDF membranes (PerkinElmer Life Sciences, Inc., Waltham, Massachusetts, USA). The membrane was incubated in 5% skim milk in PBS with 0.1% Tween 20 (PBS-T) for 1 h at room temperature, and then was incubated overnight at 4 °C in rabbit anti-human DLL3 antibody (Funakoshi Co. Ltd., 1:1000) on a horizontal shaker. Following three washes with PBS-T, the membrane was incubated with secondary antibodies at room temperature for 1 h and washed with PBS-T. The chemiluminescent reaction was carried out using ECL plus Western Blotting Detection Reagents (GE Healthcare UK, UK). Signals were detected with a Fusion FX (Vilber Lourmat, France).

### qRT-PCR

Total RNA was extracted using the RNeasy mini kit (Qiagen, Germany) according to the manufacturer’s instructions. One microgram of total RNA was subjected to qRT-PCR with the One Step PrimeScript^TM^ RT-PCR Kit (Takara Bio Inc., Japan) on the Applied Biosystems 7500 Real-Time PCR System (Applied Biosystems, Waltham, Massachusetts, USA). Predesigned Taqman fluorogenic probes and primer sets for *DLL3* (Hs01085096) and *GAPDH* (Hs03929097) were purchased from Applied Biosystems. The qRT-PCR conditions were as follows: 42 °C for 5 min and 95 °C for 10 sec, followed by 40 cycles of 95 °C for 5 sec and 60 °C for 30 sec. Relative quantification of *DLL3* was normalized to the expression of the housekeeping gene *GAPDH* with the ∆∆Ct method, and the data were expressed as the means ± standard errors. Self-designed primers and probes (Applied Biosystems) were as follows: for *HBx* expression in HepG2 and HepG2.2.15 cells, sense 5′-TGCCTTCTGACTTCTTTCCTTCC-3′, antisense 5′-GCCTGAGTGCAGTATGGTGAG-3′, probe 5′-FAM-CGAACAATGCTCAGGAGACTCTAAGGCTTC-3′, with a target fragment size of 114 bp; for *HBx* expression in HepG2 cells, HepG2 cells transfected with pGFP-HBx and Hep3B cells, sense 5′-AGGTCTTGCCCAAGCTCTTAC-3′, antisense 5′-CCCAACTCCTCCCAGTCTTTAA-3′, probe 5′-FAM-TGGACTCTCAGCAATGTCAACAACCGACCT-TAMRA-3′, with a target fragment size of 112 bp.

### Immunocytochemistry

Cell lines were fixed with 4% paraformaldehyde in PBS for 10 min, blocked for 30 min in Blocking ACE (DS Pharma Biomedical Co. Ltd., Japan), and incubated with the anti-DLL3 polyclonal antibody (Funakoshi Co. Ltd., 1:1000) for 1 h at room temperature. The cells were washed three times in PBS, incubated with goat anti-rabbit Alexa 488 (Invitrogen Life Technologies) for 1 h, and counterstained with VECTA SHIELD H1500 with DAPI (VECTOR Laboratories, Burlingame, California, USA). The cells were visualized using a laser scanning microscope (Leica TCS SP8, Germany), and images were captured with LAS X software (Leica).

### Knockdown of HBx

The pre-designed siRNAs specific for *HBx* were purchased from Invitrogen Life Technologies (USA). siRNA sequences for *HBx* were as follows: siHBx260 5′-GAAUGUUGCCCAAGGUCUUACAUAA-3′ and 5′-UUAUGU AAGACCUUGGGCAACAUUC-3′; siHBx371 5′-GGGAGGAGAUUAGAUUAA AGGUCUU-3′ and 5′-AAGACCUUUAAUCUAAUCUCCUCCC-3′; fluorescein-tagged siHBx371 was as follows: 5′-alexa488-GGGAGGAGAUUAGAUUAAAGGUCUU-3′ and 5′-AAGACCUUUAAUCUAAUCUCCUCCC-3′. HepG2.2.15 cells were plated at 20–30% confluency 24 h before transfection, and were transfected with either 1 nM *HBx* siRNA or a negative control siRNA (Invitrogen Life Technologies) using Lipofectamine RNAiMAX Reagent (Invitrogen Life Technologies) according to the manufacturer’s protocol. After 48 h of transfection, the cells were harvested and subjected to further analysis.

### Overexpression of HBx

HepG2 cells were plated at 20–30% confluency 24 h before transfection, and were transfected with either pGFP-HBx (Addgene, #24931, Cambridge, Massachusetts, USA) or control empty vector using Lipofectamine 2000 Reagent (Invitrogen Life Technologies). After 48 h of transfection, the cells were harvested and subjected to further analysis.

### Treatment with a demethylating agent and histone methyltransferase inhibitors

HepG2 and HepG2.2.15 cells were plated at 10–15% confluence. After 24 and 72 h, 1 nM demethylating agent, 5-Aza-dC (Sigma-Aldrich) was added to HepG2.2.15 cells. For inhibition of histone deacetylation, 1 nM TSA (Sigma-Aldrich) was added 72 h after plating. Total RNA was extracted using the RNeasy mini kit (Qiagen) according to the manufacturer’s instructions 96 h after plating, and qRT-PCR was performed.

### Statistical analysis

JMP 13.0 software (SAS Institute Inc., Cary, North Carolina, USA) was used for statistical analysis. Data were expressed as the mean ± standard error. The relationship between DLL3 expression and clinicopathological features or the Ishak score was analyzed with Pearson’s correlation. Statistical significance of the other data was determined with one-way analysis of variance, the Fisher’s test, or Student’s t test. Values of p < 0.05 were considered statistically significant.

### Ethics approval and consent to participate

All human samples in this study were obtained after receiving written consent from the patients.

### Consent for publication

All authors have reviewed the manuscript and consented to publication.

### Availability of data and material

All data generated or analyzed during this study are included in this published article and its additional files.

## Electronic supplementary material

Supplementary Information
