# Supplementary material for: Delta-like 3 is silenced by HBx via histone acetylation in HBV-associated HCCs
Source: Sci Rep. 2018 Mar 19;8:4842. doi: 10.1038/s41598-018-23318-1 (PMC5859037; doi:10.1038/s41598-018-23318-1)
Supplement: Supplementary file 1 — Supplementary Information [file 41598_2018_23318_MOESM1_ESM.docx]

**Delta-like 3 is silenced by HBx via histone acetylation in HBV-associated HCCs**

Hiroki Hamamoto^1^, Kentaro Maemura^2^, Kentaro Matsuo^1^, Kohei Taniguchi^1^, Yoshihisa Tanaka^2^, Sugiko Futaki^2^, Atsushi Takeshita^3^, Akira Asai^4^, Michihiro Hayashi^1^, Yoshinobu Hirose^3^, Yoichi Kondo^2^, and Kazuhisa Uchiyama^1^

Departments of ^1^General and Gastroenterological Surgery, ^2^Anatomy and Cell Biology, ^3^Pathology, and ^4^Second Department of Internal Medicine, Osaka Medical College, Takatsuki 569-8686, Japan

*Corresponding author:* Hiroki Hamamoto, MD

Department of General and Gastroenterological Surgery, Osaka Medical College, 2-7 Daigaku-machi, Takatsuki 569-8686, Japan

E-mail: [sur154@osaka-med.ac.jp](mailto:sur154@osaka-med.ac.jp)

Telephone: +81-72-638-1221

**Supplementary Table S1. Preoperative laboratory data of 10 patients**

|  | Case1 | Case2 | Case3 | Case4 | Case5 | Case6 | Case7 | Case8 | Case9 | Case10 |
| --- | --- | --- | --- | --- | --- | --- | --- | --- | --- | --- |
| Plt  (10^3^/µL) | 20.5 | 19.8 | 24.0 | 13.7 | 18.8 | 19.8 | 29.4 | 44.0 | 16.7 | 18.6 |
| AST  (U/L) | 26 | 25 | 18 | 23 | 18 | 25 | 19 | 18 | 18 | 25 |
| ALT  (U/L) | 18 | 19 | 24 | 19 | 16 | 19 | 12 | 22 | 12 | 10 |
| Albumin (g/dL) | 4.2 | 4.5 | 4.5 | 4.2 | 3.6 | 4.5 | 3.7 | 3.5 | 3.9 | 4.0 |
| T-Bil  (mg/dL) | 0.4 | 0.7 | 0.9 | 0.9 | 0.3 | 0.7 | 0.6 | 0.3 | 0.4 | 0.5 |
| PT  (sec) | 11.4 | 11.1 | 11.8 | 11.4 | 11.4 | 11.1 | 11.3 | 13.3 | 12.1 | 11.6 |
| APTT  (sec) | 32.3 | 35.1 | 30.4 | 33.0 | 28.9 | 35.1 | 31.3 | 33.6 | 38.1 | 32.2 |
| Fibrinogen  (mg/dL) | 480 | 373 | 307 | 424 | 581 | 373 | 492 | 683 | 459 | 417 |
| Antthrombin III (%) | 98 | 111 | 111 | 94 | 100 | 111 | 97 | 81 | 98 | 101 |
| Hyaluronic acid (ng/mL) | 83 | 52 | 12 | 82 | 36 | 52 | 98 | 36 | 32 | 28 |
| Procollagen Ⅲ peptide  (U/mL) | 0.71 | 0.64 | 0.58 | 0.63 | 0.76 | 0.64 | 0.80 | 1.4 | 0.54 | 0.63 |
| Type Ⅳ  collagen  (mg/dL) | 241 | 113 | 118 | 150 | 205 | 113 | 126 | 249 | 153 | 139 |

**Supplementary Figure S2. DLL3 expression in normally functioning livers**

**
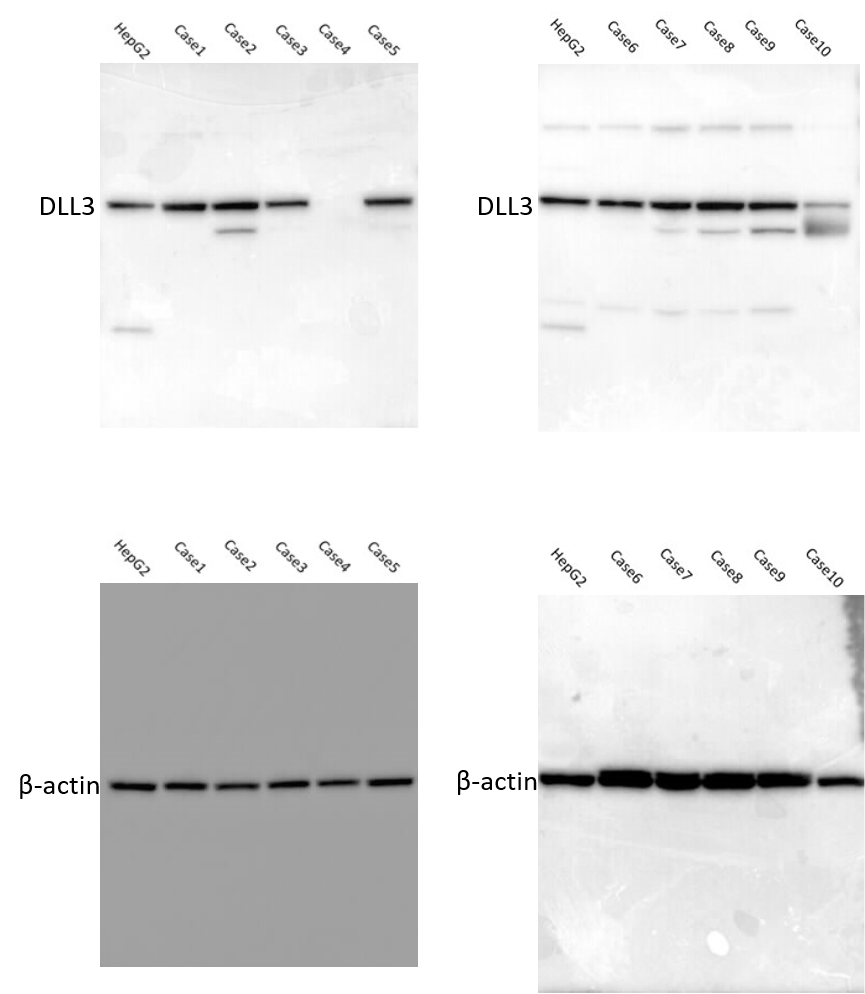
**

**Supplementary Table S3. Clinicopathological features of 46 HCC patients**

|  |  |
| --- | --- |
| Gender |  |
| Male | 35 |
| Female | 11 |
| Age (years) | 67 (52-83) |
| Virus |  |
| HBV | 11 |
| HCV | 20 |
| NBNC | 15 |
| Alcohol history |  |
| Present | 2 |
| Absent | 44 |
| Cirrhosis |  |
| Present | 17 |
| Absent | 29 |
| Differentiation |  |
| Well-Moderate | 45 |
| Poor | 1 |
| Child pugh |  |
| A | 41 |
| B | 5 |
| T stage (UICC) |  |
| 1 /2 /3 /4 | 2 /16 /22 /6 |
| Stage (UICC) |  |
| 1 /2 /3 /4 | 2 /16 /20 /8 |

HBV = hepatitis B virus

HCV = hepatitis C virus

NBNC = non hepatitis B virus and non hepatitis C virus

UICC = The Union of International Cancer Control

**Supplementary Table S4.** **Summary of Ishak score of 46 adjacent non-cancerous livers**

|  |  | Median (range) |
| --- | --- | --- |
| Necroinflammatory Scores | Interface Hepatitis | 1 (0-2) |
|  | Confluent Necrosis | 0 (0-2) |
|  | Focal Lytic Necrosis, Apoptosis,  and Focal Inflammation | 1 (0-3) |
|  | Portal Inflammation | 1 (0-4) |
| Fibrosis score |  | 3 (0-6) |

**Supplementary Figure S5. HBx expression in 10 patients with HCCs and non-cancerous livers associated with HBV**

**
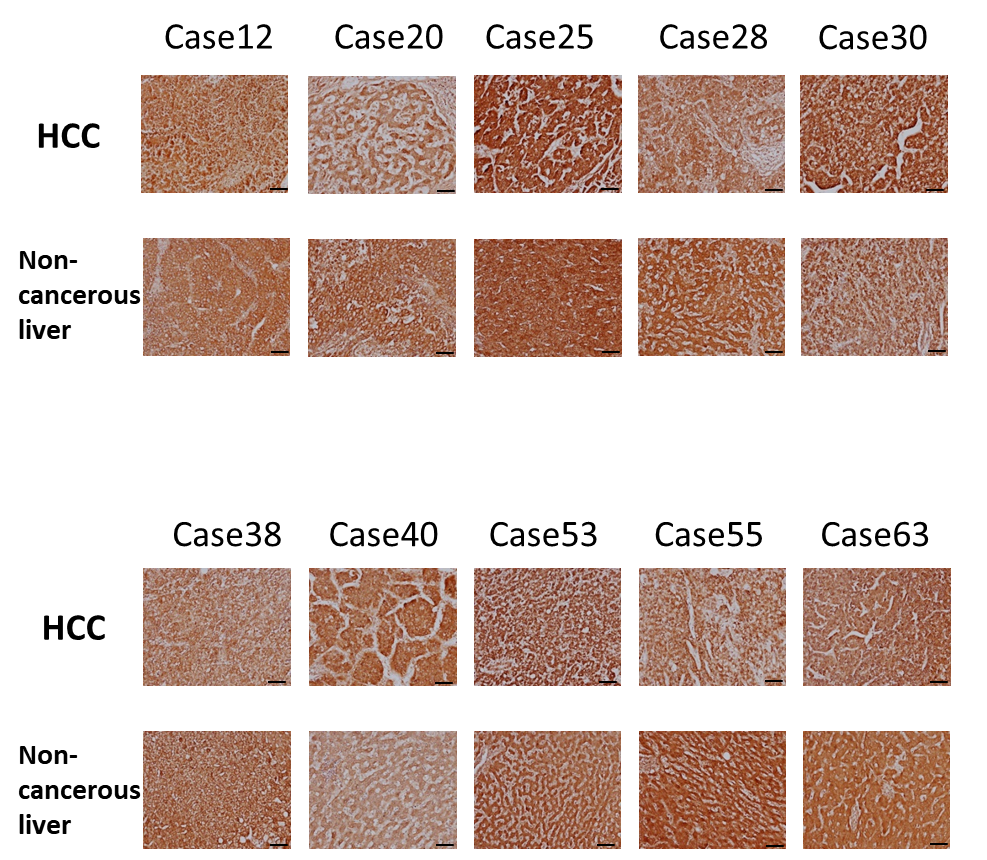
**

**Supplementary Figure S6. DLL3 expression in HBV- or HCV-associated HCCs**

**
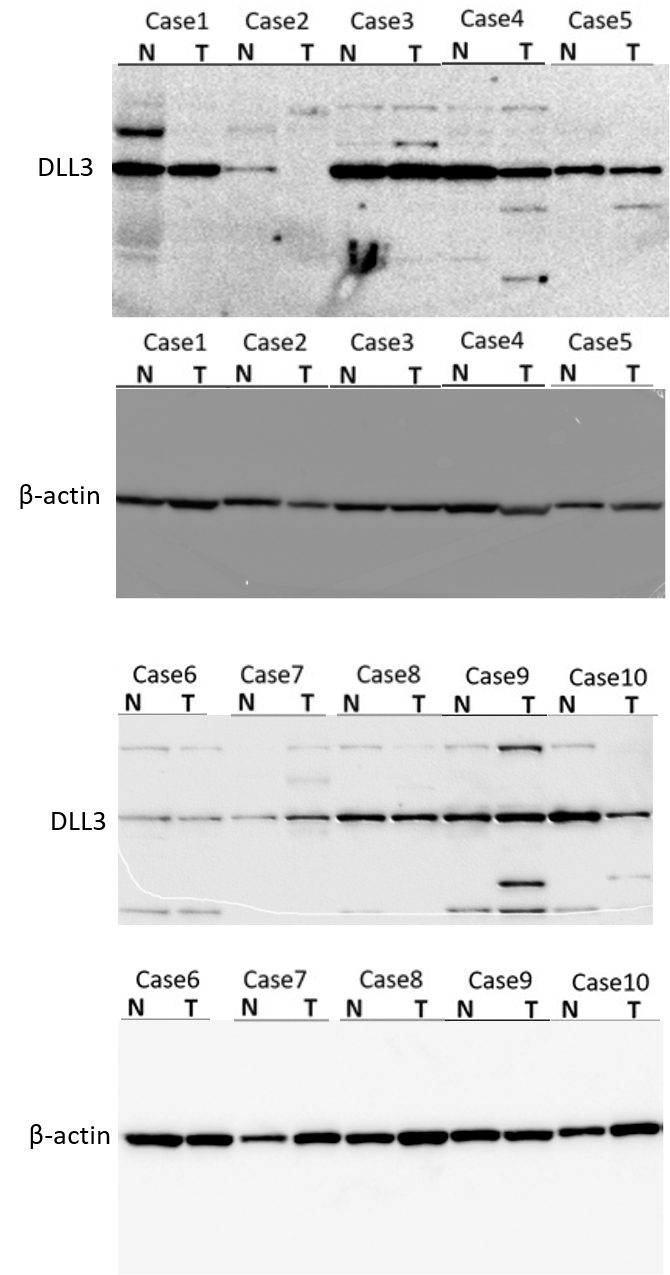
**

**Supplementary Figure S7. DLL3 expression in HepG2 and HepG2.2.15 cells**

**(a)**

**
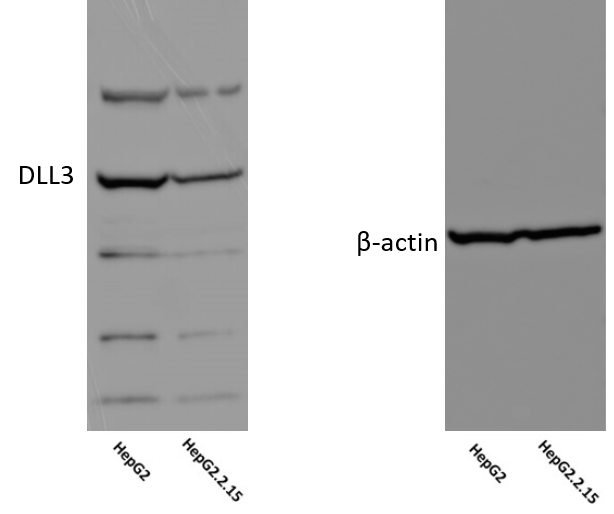
**

**(b)**

**
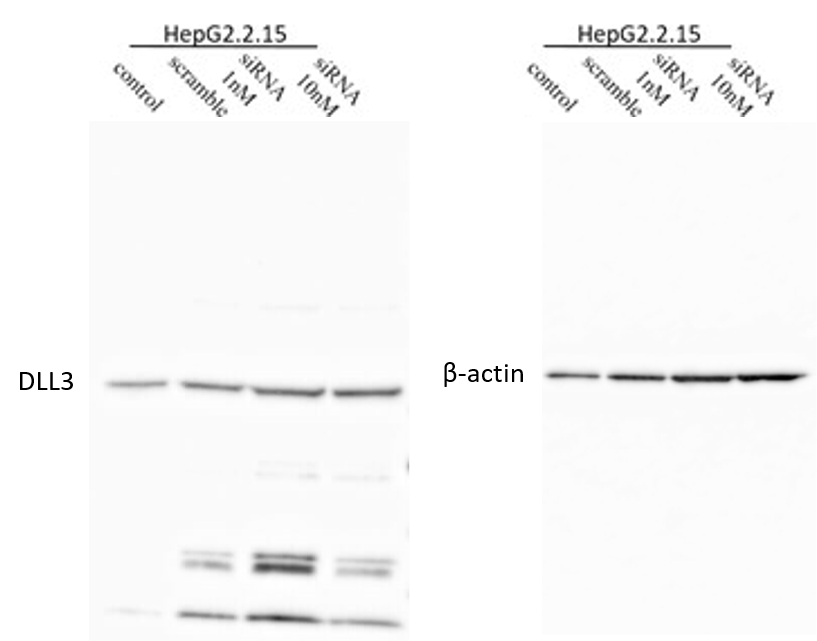
**

**Supplementary Figure S8. Gene silencing of *DLL3* by HBx siRNA**

**
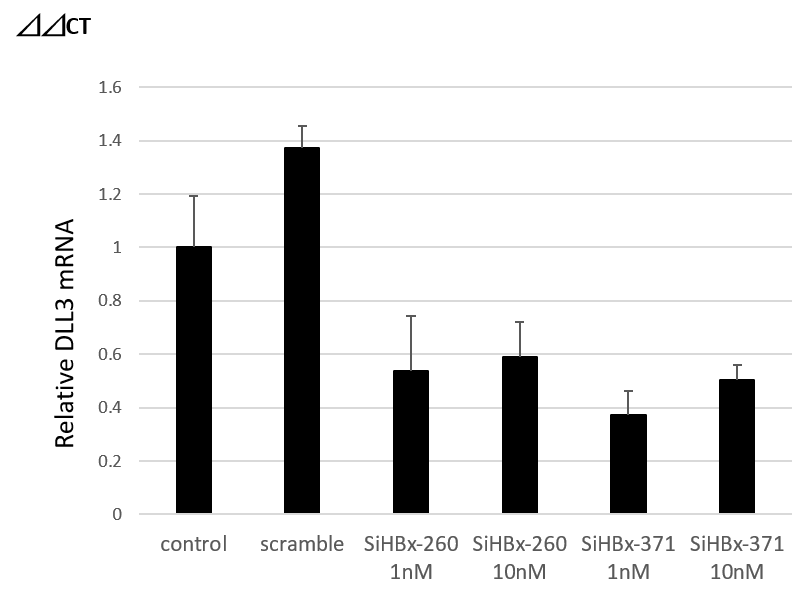
**

**Supplementary Figure legends**

**Supplementary Figure S2.**

Full blots of Figure 1b.

**Supplementary Figure S5.**

Immunohistochemical staining of HBx protein in 10 patients with HBV-associated HCC. Positive signals were detected in each HCC and non-cancerous liver. Scale bar, 100 µm.

**Supplementary Figure S6.**

Full blots of Figure 3c

**Supplementary Figure S7.**

(a) Full blots of Figure 4d. (b) Full blots of Figure 4g.

**Supplementary Figure S8.**

Relative quantity of *DLL3* mRNA in HepG2.2.15 cells treated with siHBx-260 (1 nM or 10 nM) or siHBx-371 (1 nM or 10 nM) was evaluated with qRT-PCR. siHBx-371 suppressed *DLL3* expression more strongly than siHBx-260.
